# Inferior Caballeronia symbiont lacks conserved symbiosis genes

**DOI:** 10.1099/mgen.0.001333

**Published:** 2024-12-16

**Authors:** Kaisy Martinez, Patrick T. Stillson, Alison Ravenscraft

**Affiliations:** 1 Department of Biology, The University of Texas at Arlington, Arlington, TX, USA

**Keywords:** Burkholderiaceae, *Caballeronia*, Heteroptera, iPBE, *Leptoglossus*, Pentatomomorpha, symbiosis

## Abstract

Pentatomomorphan bugs can form symbiotic associations with bacteria belonging to the supergenus *Burkholderia sensu lato*. This relationship has become a model for understanding environmental symbiont acquisition. Host insects can utilize various symbiont strains from across *Burkholderia sensu lato*; however, host colonization success and benefits conferred vary by bacterial clade. Therefore, we conducted a meta-analysis aimed at identifying candidate genes that underpin beneficial symbioses within this system. We scanned the entire Burkholderiaceae family for the presence of 17 colonization-associated genes, as well as 88 candidate genes that are differentially expressed during symbiosis. There was no difference in the distribution of the 17 colonization-associated genes between symbiotic (*Caballeronia* and insect-associated plant beneficial and environmental clade) and non-symbiotic lineages; however, there was a higher prevalence of the 88 candidate genes in the insect symbiont lineages. We subsequently analysed the genomes of nine symbiotic *Caballeronia* species that confer varying fitness benefits to their insect hosts. One symbiont species was significantly worse, one was significantly better and the remaining seven were intermediate in terms of conferred host fitness benefits. We found that species possessing a higher number of the candidate genes conferred faster host development time. Furthermore, we identified two candidate genes that were missing in the least beneficial species but present in the other eight, suggesting that these genes may be important in modulating symbiont quality. Our study suggests that the mechanisms required for host colonization are broadly distributed across Burkholderiaceae, but the genes that determine symbiont quality are more prevalent in insect-associated species. This work helps to identify genes that influence this highly specialized yet diverse symbiosis between Pentatomomorphan insects and Burkholderiaceae bacteria.

## Data Summary

Supplementary information and figures are available as additional files.

The accession numbers for the genomes used can be found in Table S1. Data used by Hunter et al. can be accessed on Dryad (https://doi.org/10.5061/dryad.2bvq83bqz). Data by Stillson et al. in review can be accessed on Dryad (https://doi.org/10.5061/dryad.zs7h44jjj).

## Introduction

Animals are highly dependent on micro-organisms, with instances of microbial symbioses observed between all domains of life [1,2]. The mode by which an animal host acquires its symbiotic partner has major implications for both the genetic architecture and the physiological, ecological and evolutionary outcomes of the relationship. Symbionts which are transmitted vertically from parent to offspring often undergo genomic reduction: their genomes shrink as genes unrelated to symbiosis are removed over evolutionary time [3]. In contrast, environmentally acquired symbionts retain their genomic features, including functions that could be detrimental to the host such as pathogenesis. Despite this, many animals still acquire their microbial partners from their local environment. For example, mosquitoes and butterflies have no microbiota upon hatching and acquire microbes as they feed; these bacteria can contribute to host nutrition or immunity, improving host fitness [4,9]. Humans, on the other hand, vertically transmit microbes during pre- and post-natal development but undergo drastic diversification during the first 3 years of life, with ~20% of microbiome diversity associated with environmental factors [10,11]. This raises the question: what genes enable environmental microbes to colonize and benefit animal hosts?

Pentatomomorphan insects in the hemipteran superfamilies Coreoidea, Lygaeoidea and Pyrrhocoroidea have evolved a highly specialized relationship with bacteria in the clade *Burkholderia sensu lato* [12,14]. Most insects are predominantly colonized by species in the *Caballeronia* genus. This symbiosis is a developing model system for environmentally acquired symbioses. The symbiont colonizes crypts in a specialized posterior midgut segment, called the M4. There, it recycles nitrogenous waste and synthesizes B vitamins for the insect. Hosts that fail to acquire the symbiont exhibit severe developmental delays, decreased body size and high mortality [15,17]. However, the symbiont is not passed from mother to progeny; instead, newly hatched nymphs must acquire it from the environment [18].

Insects that associate with *Caballeronia* have evolved selective mechanisms to sort out potential symbionts from other environmental microbes. Preceding the symbiotic M4 organ is a narrow segment of the gut, called the constricted region. This region is plugged with a mucus matrix that prevents the entrance of food and most non-symbiont species into the M4 [19]. To obtain access to the symbiotic organ, microbes must be capable of traversing the mucus matrix, which is achieved by manoeuvring their flagellum in a corkscrew motion [19,20]. Hosts also filter the symbiont pool via immune defences. Uncolonized hosts upregulate the production of antimicrobial peptides and lysosomes, suggesting that immune defences may be employed to prevent colonization from non-symbiont species [21]. Another subset of antimicrobial peptides, termed crypt-specific cysteine-rich peptides, CCRs, have been observed to selectively affect non-symbionts; resistance to these CCRs has been proposed as a crucial feature of symbiotic *Burkholderia* [22].

In the bug*–Caballeronia* model, successful symbiont colonization is characterized by penetration of the constricted region and rapid proliferation in the midgut crypts of the symbiotic organ, which has been documented in the host *Riptortus pedestris* (Alydidae) [23]. Insects can associate with several genera throughout the Burkholderiaceae family with varying levels of efficiency; associations most commonly occur with *Burkholderia sensu lato*, which is composed of three primary genera. *Caballeronia* is the most common symbiont genus, consistently achieving the highest host colonization rates (80–100%), densely filling the M4 crypts and conferring the greatest fitness benefits in terms of survival, host size, weight and growth rate [15,24]. *Paraburkholderia* primarily consists of plant-associated bacteria, including legume symbionts [25], but in *R. pedestris,* they provide intermediate host benefits with variable colonization rates (0–100%) and lower crypt density. There is an insect-associated *Paraburkholderia* subclade referred to as the ‘insect-associated plant beneficial and environmental clade’ (iPBE), which partners with largid bugs (Largidae) [13]. This clade achieved high colonization rates (70–100%) in *R. pedestris* but poorly filled the midgut crypts, suggesting a potential lack of machinery to fully colonize the host. Finally, the genus *Burkholderia sensu stricto* contains pathogenic bacteria and has consistently demonstrated low colonization rates (0–40%) with no crypt development [24] but has been detected in overwintering *R. pedestris* [26].

In general, host insects benefit when colonized by any *Caballeronia* strain compared to hosts lacking a symbiont [15,16, 24]. However, some *Caballeronia* strains confer higher insect fitness: one study found that insects colonized by one *Caballeronia* species developed slower and weighed less than hosts colonized by three other *Caballeronia* species [16]. What mechanisms drive these differential outcomes among *Caballeronia* remains an open question.

To better understand the genetic mechanisms underlying these symbiotic relationships, we performed a literature review to compile a list of candidate bacterial genes that may be involved in host colonization, symbiont proliferation within the M4 and/or symbiont influence on host performance. These included 1165 genes that were differentially expressed during symbiosis and were involved with the assimilation of some sugars, degradation of fatty acids, transport and assimilation of host nitrogenous wastes and more [27]; 282 genes that were conserved in insect-associated *Caballeronia* and iPBE genomes and absent from non-symbiotic species [13] and 17 genes gathered from various loss of function studies that are involved in symbiont-host signalling to increase oxygen to the midgut, symbiont stress response, oligosaccharide O-antigen modification, purine biosynthesis, biofilm formation, cell wall modification and flagellar modification [19,28,34]. We then compared these genes' presence across a broad diversity of Burkholderiaceae genomes to reveal the genetic commonalities and differences between symbiotic and non-symbiotic Burkholderiaceae lineages. Our goal was to generate a list of potentially important symbiosis-associated genes to improve our ability to predict symbiotic outcomes and to help direct future research on the genetic underpinnings of this symbiosis.

## Methods

### Identification of orthologous genes

**Fig. 1.**
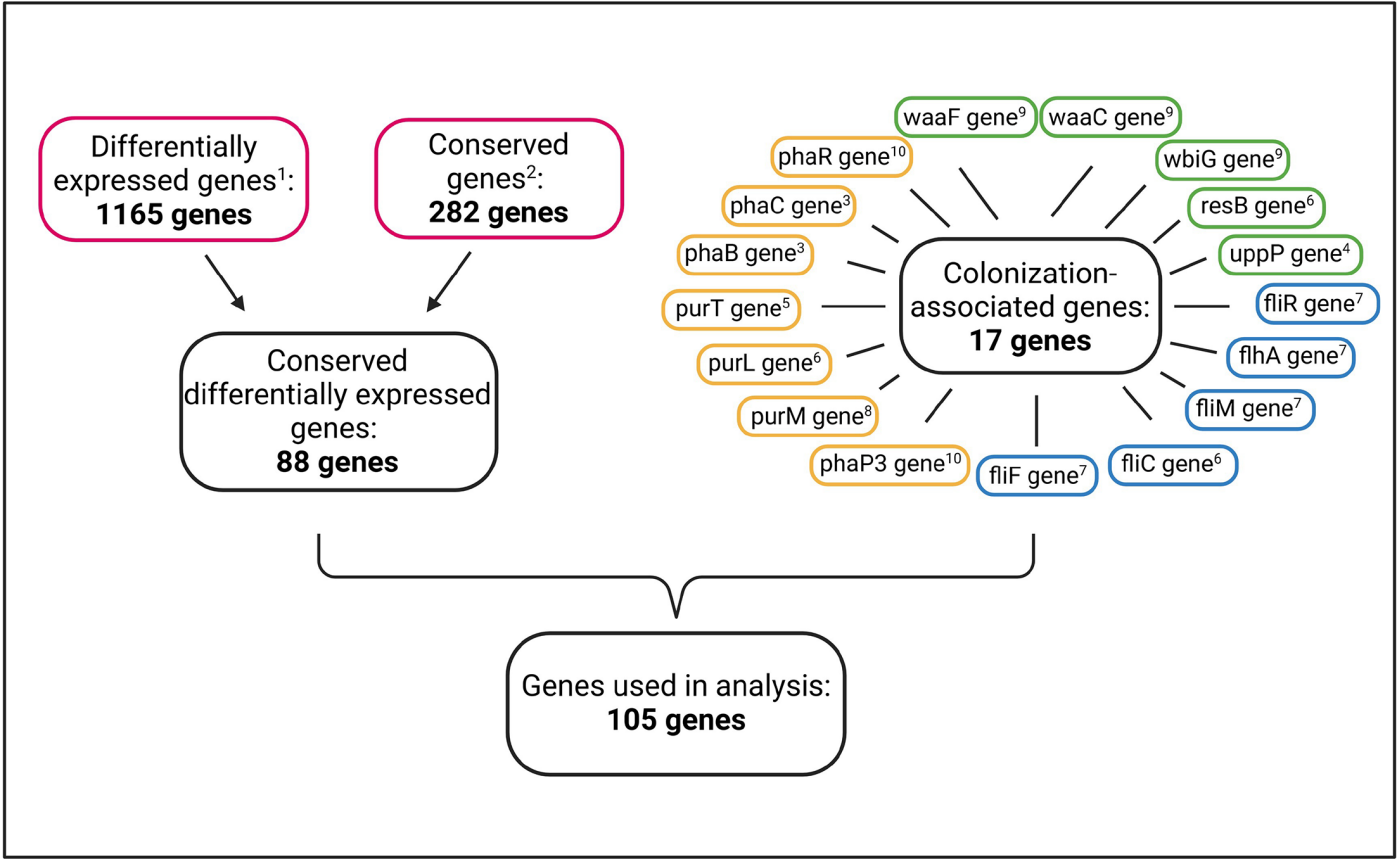
Origin of genes used in analyses. Through transcriptomics, Ohbayashi *et al.* identified 1165 genes that were differentially expressed by *Caballeronia* during symbiosis compared to in culture. Through comparative genome analysis, Takeshita and Kikuchi identified 282 genes that were conserved in symbiotic species. These two studies, shown in red, share 88 genes. An additional 17 colonization-associated genes were identified from several knock-out studies. These genes are colour-coded by functional groups, with green indicating cell wall components, yellow for stress response and blue for motility. Citations: 1. Ohbayashi *et al*. [27], 2. Takeshita and Kikuchi [13], 3. Kim *et al*. [33], 4. Kim *et al*. [30], 5. Kim *et al*. [32], 6. Jang *et al*. [29], 7. Ohbayashi *et al*. [19], 8. Kim *et al*. [31], 9. Kim *et al*. [30] and 10. Jang *et al*. [28].

We performed a literature review to identify all *Caballeronia* genes associated with symbiosis. There were 88 genes that were both conserved across three *Caballeronia* and two iPBE species and were found to be differentially expressed during symbiosis [13,27]. We termed these ‘candidate symbiosis genes’. Additionally, we compiled a list of 17 genes shown to be associated with symbiont colonization of the host M4 midgut region [13,19, 28,34], which we refer to as ‘colonization-associated genes’ (Fig. 1).

To identify if the 88 candidate genes and the 17 colonization-associated genes had orthologs across the Burkholderiaceae family, blastX was performed via the National Center for Biotechnology Information (NCBI) on 333 genomes from 21 genera [35,36]. The blasted gene sequences for a documented symbiosis-associated genomic region [13,37] were extracted from *Caballeronia zhejiangensis* (accession: GCA_022879815) using the same gene accessions as Stillson *et al*. [37], while the remaining sequences were extracted from *C. insecticola* (accession: GCA_000402035) using the gene accessions provided in Ohbayashi *et al*. [27]. Following the ortholog criteria established by Stillson *et al*. [37], search results with an E-value ≤e^–20^ and query coverage ≥70% were considered strong ortholog candidates. Potential ortholog candidates were also considered if they met two of the three following criteria: query protein had the same name as the searched protein, an E-value ≤e^–3^ or query coverage ≥30%.

We evaluated whether these genes are associated with any specific *Burkholderia* s.l. genus or if they are broadly distributed across all Burkholderiaceae species. This was done by comparing the upregulated genes (more highly expressed during symbiosis compared to the free-living state), downregulated genes (less expressed during symbiosis) and colonization-associated genes possessed by each genus using linear models. In the three models (total upregulated candidate gene count, total downregulated candidate gene count and total colonization-associated gene count), the gene counts were considered the response variable, while the genus was set as the fixed effect (i.e. *Caballeronia*, *Paraburkholderia*, *Burkholderia* s.s., the iPBE clade and all other non-*Burkholderia* species).

### Protein structure and function

The 88 candidate genes included 6 novel hypothetical proteins of unknown function detected only in the Burkholderiaceae family. To infer their functions, we used Alphafold 2 v.2.1.2 [38] to predict the most likely protein conformation based on the amino acid sequences. This was paired with ProteInfer v.1 [39] to identify the most likely pathways that these hypothetical proteins would be associated with by using deep convolutional neural networks to predict protein function.

### Phylogenetic analysis

We built two phylogenies to determine the distribution of our focal genes within the Burkholderiaceae family and the *Caballeronia* genus. Genomes for 333 species (86 *Caballeronia*) were downloaded from the NCBI GenBank database (all unique complete genomes as of March 2023) to construct whole-genome-based phylogenies (Table S1, available in the online Supplementary Material). *Bordetella bronchiseptica* was selected as the outgroup for the family phylogeny and *Trinickia symbiotica* was selected for the *Caballeronia* genus phylogeny. The phylogenies were constructed using Realphy [40] with a gap threshold of ‘0.1’, with *Burkholderia cepacia* and *C. insecticola*, respectively, selected as the reference genomes. Realphy uses Bowtie2 v2.0.0 [41] to map each genome to the reference species' genome followed up with PhyML v3.0 to construct the most-likely phylogenetic tree [42]. The phylogeny was visualized with ape v5.4–1 [43].

Phylogenetic signal was evaluated with Moran's I (function = ‘abouheif.moran’; package = ‘adephylo’) and Pagel's λ (function = ‘phylosig’; package = ‘phytools’) to identify whether the variation in symbiont gene count across the *Caballeronia* genus could be predicted under a Brownian motion model, suggesting whether symbiont relatedness may be associated with symbiont-conferred host fitness.

### Strain variation on host fitness

To test for correlations between candidate genes and symbiont effects on host fitness, we evaluated previously collected data on host weight and development time for two congeneric host insect species (*Leptoglossus phyllopus* and *Leptoglossus zonatus*) reared at 28 °C with nine symbiont strains (Table 1) ([16]; Stillson *et al*., *in review*). Final host weight at adulthood and host development time from the third instar (development stage) to adulthood were used as proxies for host fitness. The effect of symbiont strain on host fitness was evaluated using linear mixed-effect models, with weight or development time as the response variable and symbiont strain and host species as fixed effects. Sex was included as a fixed effect in the weight model to account for females being larger than males [16]. Data from Stillson *et al*. can be found on Dryad (https://doi.org/10.5061/dryad.zs7h44jjj). All statistical analyses were conducted in R v.4.2.2 [44].

**Table 1.** *Caballeronia* strains with measured host fitness effects. Host insects and the sample sizes used in the studies are reported for each *Caballeronia* strain. When both species were measured, the respective sample sizes for each species were listed in parentheses

*Caballeronia* strain	Host species	Sample size
Lep1A1	Leptoglossus phyllopus* , Leptoglossus zonatus*	35 (19, 16)
Lep1P3	Leptoglossus phyllopus* , Leptoglossus zonatus*	46 (23, 23)
LP003	*Leptoglossus phyllopus*†	18
LZ003	*Leptoglossus phyllopus*†	39
LZ008	*Leptoglossus phyllopus*†	34
LZ019	*Leptoglossus phyllopus*†	22
LZ062	*Leptoglossus phyllopus*†	20
SL2Y3	Leptoglossus phyllopus* , Leptoglossus zonatus*	46 (25, 21)
TF1N1	Leptoglossus phyllopus* **,†** , Leptoglossus zonatus*	55 (44, 11)

*Acquired from Hunter *et al*. (2022). Acquired from Stillson . ()

†Acquired from Stillson *et al*. (*in review*).

## Results

### Gene comparisons across Burkholderiaceae clades

In our survey, a total of 333 unique Burkholderiaceae genomes were collected from GenBank. These genomes spanned 21 genera, with 86 *Caballeronia* genomes, 70 *Paraburkholderia*, 9 iPBE *Paraburkholderia*, 39 *Burkholderia* s.s. and 116 genomes from the 18 remaining genera (Fig. S1). These genomes were scanned for the 88 candidate symbiosis genes as well as the 17 colonization-associated genes.

Within the 88 candidate genes, there were 27 hypothetical genes. We identified as many as possible using blastX to better infer the possible functional roles they play in the bug*–Caballeronia* symbiosis. We identified 18 of the 27 hypothetical genes as adenylate/guanylate cyclase, amidohydrolase family protein, aralkylamine dehydrogenase, cytochrome B561, diacylglycerol kinase family protein, diguanylate cyclase with PAS/PAC sensor (two copies), DUF2968 domain-containing protein, ethyl *tert*-butyl ether degradation (EthD), extracellular solute-binding protein family 3, four-carbon acid sugar kinase family protein, HEAT repeat protein, initiator RepB protein, lytic transglycosylase catalytic protein, ParB-like partition protein, periplasmic sensor signal transduction histidine kinase, serine hydrolase and transposase. Of these, 5 are upregulated during symbiosis, while the remaining 13 are downregulated. The entire list of genes present in each genome, including the expression status provided by Takeshita and Kikuchi, is listed in Table S1.

**Fig. 2.**
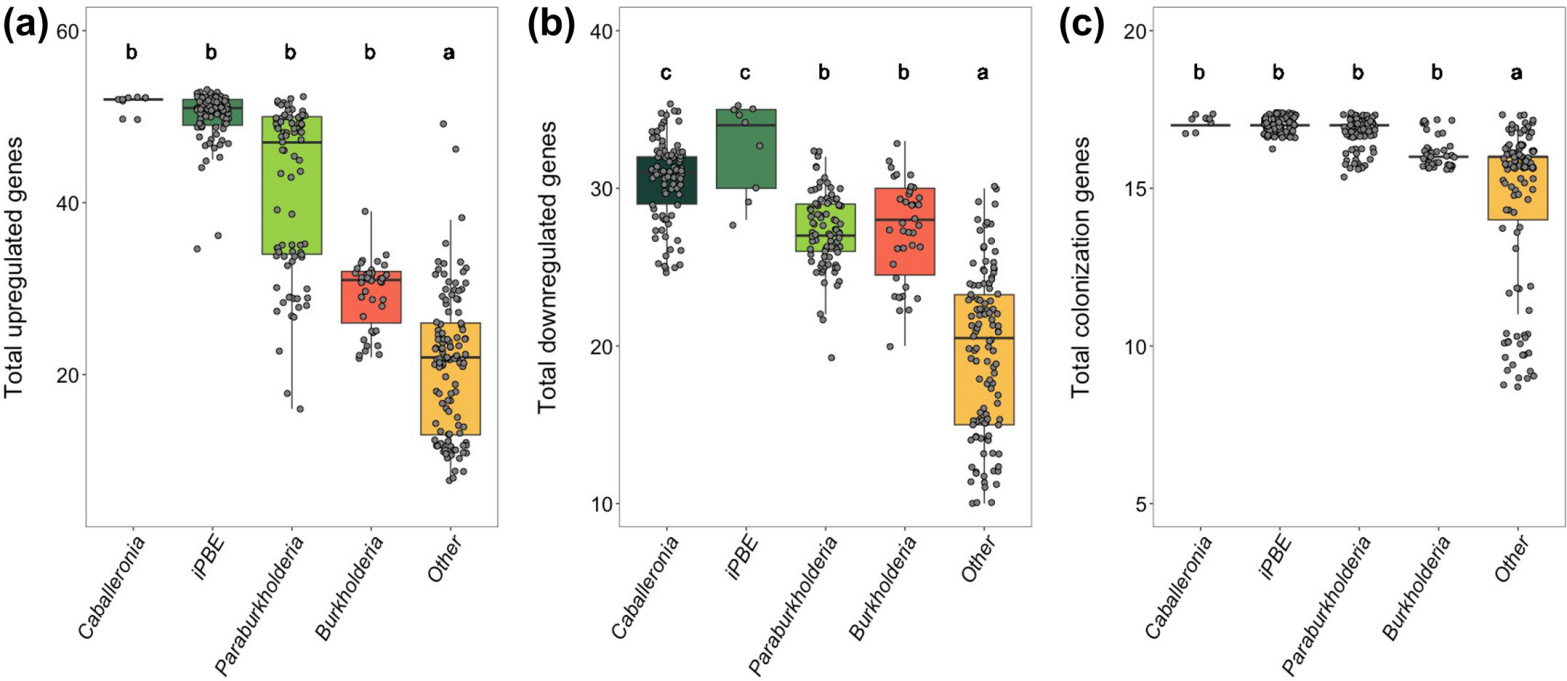
Total number of (**a**) upregulated genes, (**b**) downregulated genes and (**c**) colonization-associated genes within genomes belonging to different bacterial clades. The letters above bars denote significant differences between clades (Tukey’s honestly significant difference **(**HSD) test <0.05).

The number of candidate genes per species ranged from 20 to 88 across the entire Burkholderiaceae family, with *Caballeronia* genomes containing an average of 81 genes (86 genomes), non-iPBE *Paraburkholderia* with 70 genes and the iPBE subclade with 84 genes (90 and 9 genomes, respectively), *Burkholderia* s.s. with 56 genes (39 genomes) and the remaining species with an average of 40 genes (116 genomes). There were significant differences between the total number of upregulated candidate genes (*df*=4, *F*=241.90, *P*<0.001; Fig. 2a), downregulated candidate genes (*df*=4, *F*=124.91, *P*<0.001; Fig. 2b) and colonization-associated genes (*df*=4, *F*=38.93, *P*<0.001; Fig. 2c) among genera. Both insect-associated groups (i.e. *Caballeronia* and iPBE) had significantly more upregulated and downregulated candidate genes than *Paraburkholderia*, *Burkholderia* s.s. and the non-*Burkholderia* species. As for the colonization genes, no difference in gene count was detected across the *Burkholderia* s.l. genera, but these all possessed more genes than the non-*Burkholderia* s.l. species.

### Unique protein-coding genes

All but 6 of the 105 focal genes were detected in at least one other bacterial family (a minimum of one match for each gene met the minimum threshold set for an ortholog candidate). The six proteins unique to Burkholderiaceae (accessions: BAN24398, BAN25003, BAN27455, BAN27489, BAN27540, BAN28196) were all hypothetical protein-coding genes (not identified via blastX).

**Fig. 3.**
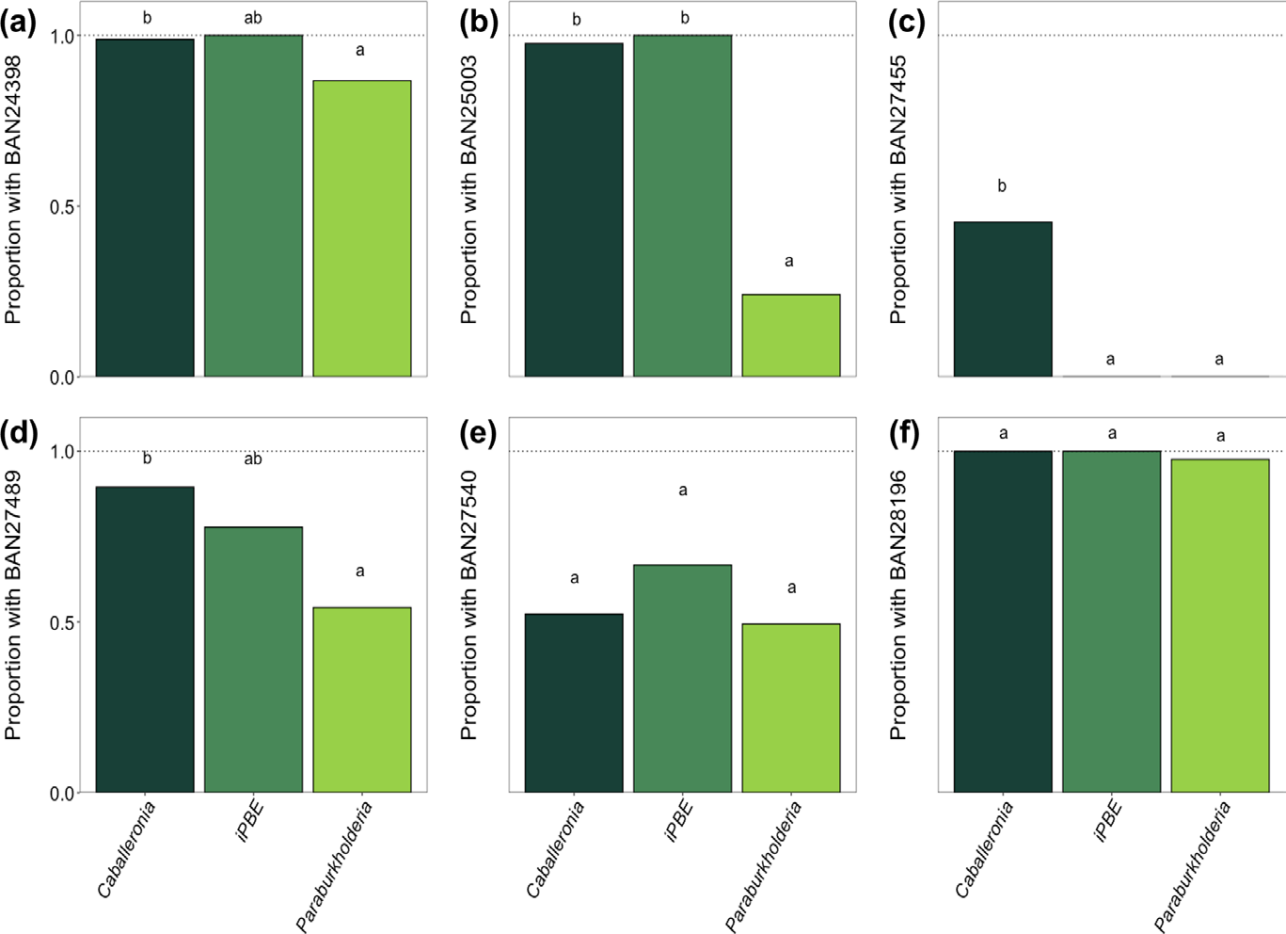
The proportion of genomes within the insect-associated clades that possess hypothetical genes (**a**) BAN24398, (**b**) BAN25003, (**c**) BAN27455, (**d**) BAN27489, (**e**) BAN27540 and (**f**) BAN28196. The letters above bars denote significant differences between clades (Tukey’s HSD <0.05).

Due to the novelty of these six genes in the Burkholderiaceae family, we further analysed their abundance across the symbiotic clades (*Caballeronia*, *Paraburkholderia* and iPBE). There was a significant difference in the proportion of genomes containing the six hypothetical genes (*df*=2, *F*=57.90, *P*<0.001; Fig. 3). BAN27455 was present more frequently in *Caballeronia* than the other clades; BAN24398, BAN25003 and BAN27489 were present more frequently in *Caballeronia* and iPBE than in *Paraburkholderia*; and the proportion of genomes possessing BAN 27540 and BAN28196 did not differ between the clades (Fig. 3).

To attempt to infer the potential functionality of these six unique hypothetical protein-coding genes, we first searched various databases (e.g. InterPro, CAZy, KEGG, EggNOG) to investigate which protein families the hypothetical proteins belong to. All searches provided no results, further supporting the novelty of these genes within the Burkholderiaceae family. We subsequently identified the most likely predicted structures and functions using Alphafold 2 and ProteInfer. BAN24398, BAN27455 and BAN28196 were predicted to be cellular components (93%, 70% and 89% likelihood, respectively), while BAN25003 is involved in cellular transport (72% likelihood), BAN27489 is involved in regulating biological processes (76% likelihood) and BAN27540 functions in nitrogen compound metabolism (100% likelihood). The predicted protein structures are shown in Fig. S2.

*Can we predict a‘good’symbiont?* To assess whether certain clades of *Caballeronia* might be better bug symbionts, we tested for phylogenetic signal in the number of candidate symbiosis genes across the genus. We found that *Caballeronia* species resemble each other slightly more than expected under a Brownian motion model, and the number of candidate symbiosis genes can be explained by relatedness between species (*I*=0.24; λ=0.99, *P*≤0.001; Fig. S3), suggesting that species that are more closely related will have similar numbers of candidate symbiosis genes.

**Fig. 4.**
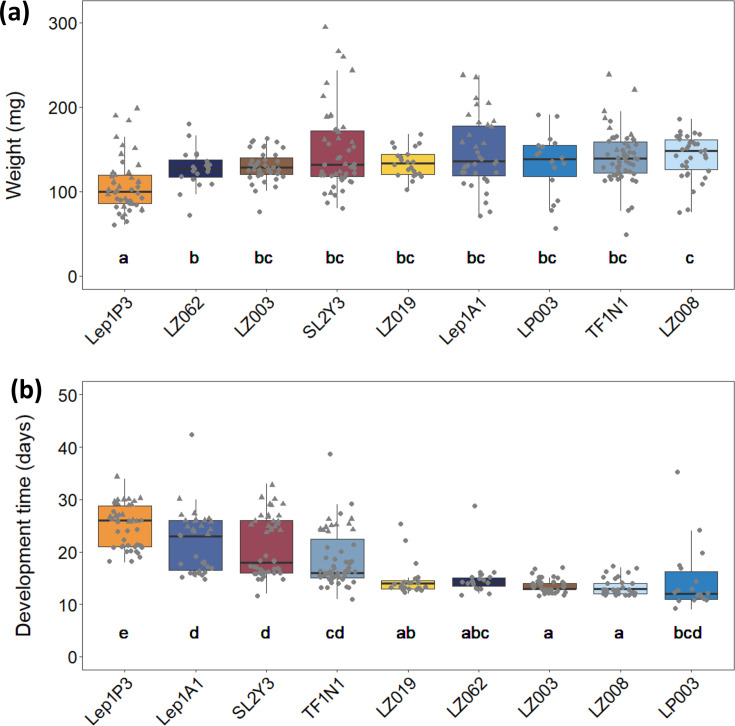
Fitness benefits conferred by different *Caballeronia* isolates to insect hosts (sorted by medians) for (**a**) weight and (**b**) development time. The letters below bars denote significant differences between strains (Tukey’s HSD <0.05).

**Fig. 5.**
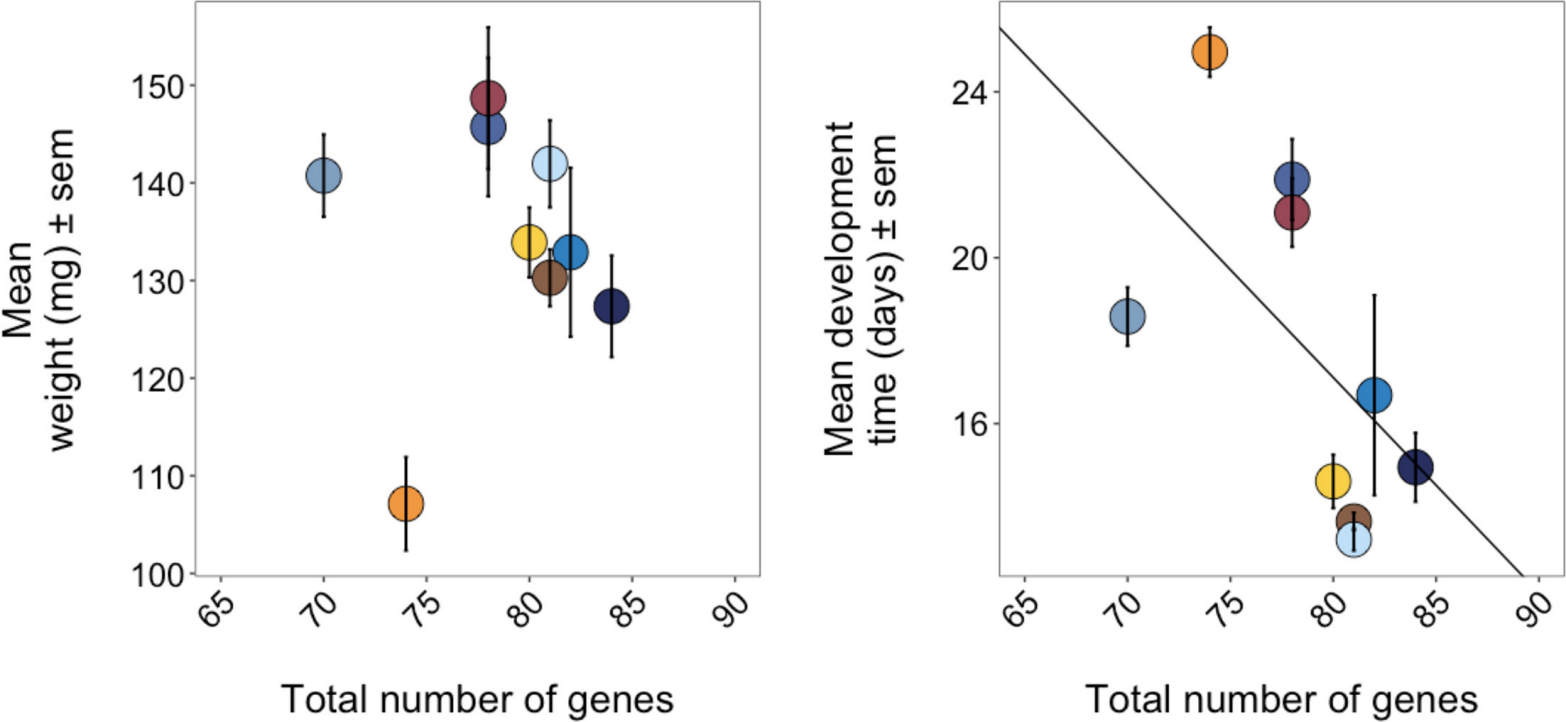
Symbiont strain-associated host fitness metrics include (**a**) total genes by mean host weight and (**b**) total genes by mean host development time. Each colour represents a single symbiont strain.

We took advantage of host fitness data from prior studies of two congeneric species of *Leptoglossus* leaf-footed bugs ([16]; Stillson *et al*., *in review*) to test for correlation between a symbiont species' number of candidate genes and the benefit it conferred to host insects. Across these studies, insects were inoculated with one of nine different *Caballeronia* strains, and their performance was recorded. We compared insect weight and development times across strains to identify if any were better or worse for the host. One strain, Lep1P3, was identified as significantly worse for both host weight (*df*=8, *F*=11.22, *P*<0.001) and development time (*df*=9, *F*=36.44, *P*<0.001), while LZ008 was the best strain for both fitness metrics. For the other seven strains, minimal differences were detected between host weights, while development times varied by strain (Fig. 4). Host weight was not correlated with total gene count (*r*=0.03, *P*=0.57; Fig. 5a), but we observed a negative correlation between a strain's number of candidate symbiosis genes and its host's development time: time to adulthood decreased by 0.52 days (or 12.48 h) for each additional symbiosis-associated gene (*r*=−0.36, *P*<0.001; Fig. 5b). There were exceptions to these trends. Moderately beneficial strain TF1N1 possessed fewer total symbiosis-associated genes than the least beneficial strain, Lep1P3 (70 and 74 genes, respectively), despite their relatedness (Fig. S3). Additionally, Lep1P3 was an exception to the trend in decreasing adult host weight with an increased quantity of genes.

We therefore asked which of the symbiosis genes were lacking in the inferior symbiont Lep1P3. This strain was missing 2/105 genes that were present in each of the other eight strains, with both genes being upregulated during symbiosis. One gene encodes acyltransferase 3 (BAN27429), while the other encodes the novel hypothetical protein BAN27489. The superior strain LZ008 possesses two genes found only in it and strain LP003, including hypothetical protein BAN27540, which is downregulated during symbiosis, and a HEAT repeating protein (BAN25772), which is upregulated during symbiosis and functions in metabolic processes.

Finally, we tested whether variation in candidate symbiotic gene count and conferred performance metrics was correlated with the phylogenetic relatedness of the *Caballeronia* strains. Candidate gene count was explained by the strain's phylogenetic relatedness (λ=1.0, *P*<0.001), but we could not rule out the possibility that symbiont phylogenetic relatedness does not fully explain conferred host outcomes (weight: λ=0.93, *P*=0.51; development: λ=0.89, *P*=0.15); in other words, the variation in conferred host weight or conferred development time may be affected by factors other than symbiont phylogenetic relatedness.

## Discussion

To better understand the mechanistic basis and evolutionary trajectory of symbiotic relationships, it is essential to identify symbiont genes that underpin these associations (e.g. those necessary for *in vivo* survival or used to synthesize compounds for the host) and to describe their distribution across microbial clades. By performing a family-wide meta-analysis of 105 candidate genes identified from the literature, we found that there are differences in the total number of candidate genes across the Burkholderiaceae family, with the *Caballeronia* and iPBE clades possessing more candidate genes than any other clade. This supported our expectations, as these two clades are the most beneficial to insect hosts [14,15, 24] and genes found more predominantly in these species may contribute to this symbiosis. Furthermore, of these 105 genes, 6 were found exclusively in the Burkholderiaceae family. These were all hypothetical protein-coding genes and most prevalent in the symbiotic clades (*Caballeronia* and iPBE). We also identified candidate genes that may be associated with improved host fitness outcomes. Two genes, acyltransferase 3 and hypothetical protein BAN27489, were upregulated in previous studies and uniquely absent in the symbiont known to confer the lowest host fitness. These two genes may encode machinery that contributes to symbiont survival or contributes to improved functionality in the host and are promising targets for manipulative experiments.

### Origin of candidate symbiosis genes

Many of the identified candidate genes are ancestral to Burkholderiaceae, with orthologous genes identified across numerous bacterial families.

Furthermore, the 17 known colonization-associated genes are distributed across *Burkholderia* s.l., suggesting that while these genes may be necessary for *Caballeronia* to successfully colonize an insect host, they pre-date the symbiosis and appear to have been co-opted for this purpose rather than evolved *de novo*. This has been observed in numerous bacteria, with motility, changes to cell membrane structures and biofilm formation being key traits used in colonizing or parasitizing both plants and animals (reviewed in [45]).

Although the presence of any specific genes may not explain all the variation in colonization success found between the different *Burkholderia* s.l. genera, the *Caballeronia* species share more candidate genes with each other than would be expected under a Brownian evolution model. Despite this, there is still variation in the conferred host weight and development time by symbiont species, suggesting other factors may be involved. This variation may be due to shifts in the functionality of some of these candidate genes or shifts in their expression, which may improve host performance, rather than this variation being caused by the presence or absence of specific gene sets.

### Candidate symbiosis genes and symbiosis

Of the remaining 88 candidate genes, 53 were reported to be upregulated during symbiosis [27]. Based on the KEGG pathways of these 53 genes, we sorted them into several top categories, including amino acid biosynthesis, antibiotic biosynthesis, antibiotic and antimicrobial peptide resistance, B-vitamin biosynthesis, biofilm formation and cellular transport. These can be more simply divided into host–symbiont and microbe–microbe interactions.

For host–symbiont interactions, the *Caballeronia* symbiont upregulates genes for amino acid and B vitamin biosynthesis to produce essential nutrients that are lacking in the host's phloem-based diet. Phloem feeders need their symbionts to produce amino acids for them, as phloem does not supply many of the essential amino acids insects require (observed in aphids and stinkbugs) [46,47]. Similarly, *Caballeronia* also upregulates genes for B vitamin biosynthesis. In general, insects cannot synthesize eight B vitamins [48]; therefore, symbionts are essential for their production. This has been observed in both obligate blood feeders, including mosquitoes, ticks, and bedbugs, as well as in phloem feeders like aphids [49,51].

There is also an increase in cellular transport and biofilm-associated genes. These genes are used not just by symbionts but also pathogens when they both colonize their hosts [45]. Cellular transport mechanisms are used by symbionts to import the resources they need from their hosts and to export essential nutrients their hosts require. This would include the symbiont export of amino acids and B vitamins. These processes are observed in insects as well as in corals [52,53]. In pathogens, these genes are used instead to parasitize the host and take host-produced resources [45]. Similarly, biofilm genes are also involved in the *Caballeronia* colonization effort (also observed in the tsetse fly-*Sodalis* system [54]), with some of these genes included in our analyses as part of the 17 colonization-associated genes [32]. This indicates that there are a number of genes involved in biofilm formation, indicative of their importance in symbiosis. Biofilms are often used by bacteria to evade the host immune response by preventing immune cells and antimicrobial compounds from reaching the bacterial cells [55].

Finally, there are upregulated genes associated with antimicrobial peptide resistance. These peptides are produced as part of the host immune response. Protection from some antimicrobial peptides is essential for the *Caballeronia* symbiont, as the bug host produces multiple peptides to protect itself from foreign bacteria. In the bug*–Caballeronia* host *R. pedestris*, they produce several peptides that are ineffective against *Caballeronia*, but these eliminate *Escherichia coli* and *Staphylococcus aureus* [56,57]. This antimicrobial peptide resistance may be important during the early stages of *Caballeronia* colonization when the symbiont is acquired from the environment along with any other microbes collected by the host in their search for a symbiont [12,19].

As for microbe–microbe interactions, the *Caballeronia* symbiont upregulates genes for antibiotic biosynthesis (specifically the neomycin, kanamycin and gentamicin biosynthesis pathways) and antibiotic resistance (specifically vancomycin and beta-lactam pathways). These general processes are common from an environmental bacteria perspective because environmental bacteria will produce antibiotics to eliminate competition, while resistance genes are necessary to protect themselves from other microbes [58]. In bug*–Caballeronia*, the production of antibiotics may eliminate non-symbiotic bacteria and could also help defend the host, while antibiotic resistance may be used to protect the *Caballeronia* symbiont from foreign bacteria during the colonization process.

### Target genes of interest

Although no genes were unique to the symbiotic groups (*Caballeronia* and iPBE), we identified six found exclusively in the Burkholderiaceae family, with *Caballeronia* tending to possess more of these novel protein-coding genes. Therefore, we speculate that these genes may have symbiosis-associated functionality. BAN24398, BAN27455 and BAN28196 were broadly found to be associated with cellular components. Due to this general cellular functionality, it is possible that these genes might contribute to the cellular remodelling that *Caballeronia* cells undergo during colonization. These changes involve alterations to cell shape, motility, cell wall structure, respiration and metabolism [27,30, 33]. BAN25003 is suspected to function in cellular transport. Symbionts require these mechanisms to collect nutrients from their host while also exporting amino acids and nutrients that the host requires [52,53]. Interestingly, one of the six genes, BAN27540, was reported to function in nitrogen metabolism – an important symbiosis-associated function. Many host–microbe symbioses hinge upon nitrogen metabolism and exchange, ranging from legume–*Rhizobium* [59], to *Paramecium–Chlorella* [60], to *Blochmania–Camponotus* [61]. *Caballeronia* is also implicated in nitrogen metabolism in true bugs, assimilating insect nitrogenous wastes (e.g. allantoin and urea) [27] to recycle host nitrogen as this is lacking in the host's diet. Acquiring sufficient nitrogen from diet alone can be challenging for herbivorous insects, so often symbionts will recycle host nitrogen. This behaviour has been observed in other insects, including aphids [62] and ants [61,63]. Although nitrogen recycling is reported to occur in bug*–Cablleronia* [27], this gene may not contribute much to this process as this gene is downregulated during symbiosis.

The inferior symbiont, Lep1P3, was missing two genes, including the sixth Burkholderiaceae-specific candidate gene BAN27489 and the acyltransferase 3 gene. We were unable to predict the function of BAN27489 with any specificity, but its absence from the inferior symbiont and its restriction to Burkholderiaceae make it interesting for follow-up. Acyltransferase 3 proteins transfer activated acyl groups across the cell membrane and have been implicated in the modification of cell membrane lipopolysaccharides such as O-antigens [64]. As O-antigens are extracellular, they are often targeted by the host immune response; thus, bacteria have developed mechanisms to modify these surface structures to evade host defences [64]. For example, in *Salmonella typhimurium*, an acyltransferase 3 domain was implicated in the acetylation of the pathogen's O-antigen group, changing its immunological effects [65]. Acyltransferase 3 and related transferases have also been identified as potentially contributing to host colonization in the legume–*Rhizobium* system by aiding in the production of an exopolysaccharide required to establish the symbiosis [66,68], with symbionts lacking this protein experiencing impaired host colonization [69]. In bug*–Caballeronia*, lipopolysaccharides and O-antigens are reported to contribute to antimicrobial peptide resistance in the hostile host midgut, with this resistance being crucial for initial colonization [22]. However, once established, free-living *Caballeronia* loses its O-antigen [24,30, 70]. The mechanism by which this occurs is still unknown; therefore, more research is needed to identify this mechanism and to evaluate whether acyltransferase 3 may be involved in this process.

### Conclusions

In our meta-analysis, we identified 105 potential symbiosis-associated genes and investigated their phylogenetic distribution across the Burkholderiaceae family to infer their potential contributions to the bug*–Caballeronia* symbiosis. Our findings indicate that the identified candidate genes are broadly distributed across the entire Burkholderiaceae family, suggesting an ancestral origin for many of them, but they may have been co-opted for symbiosis as the insect-associated clades possess the largest number of these genes. With six of these candidate genes detected in only the Burkholderiaceae family, this may be indicative of novel genes that may contribute to the success of the numerous symbionts and pathogens found throughout this family.

We further evaluated the presence of these candidate genes among a set of *Caballeronia* isolates with known host fitness data to determine if these genes could be used to predict symbiont quality. We found that symbiont species possessing a higher number of candidate genes promoted faster host development to adulthood. Furthermore, we identified three particularly promising protein-coding genes, one of which is predicted to be involved in nitrogen metabolism and restricted to the Burkholderiaceae family, and two that are lacking in an inferior symbiont strain. One of these two genes is predicted to function broadly in biological processes, while the other, acyltransferase 3, has been implicated in host colonization due to its functions in lipopolysaccharide and O-antigen modification in other symbioses. Overall, our results provide a condensed list of candidate symbiosis-associated genes as well as three key target genes for future *in vivo* knockout studies aimed at deciphering the intricate association between Pentatomomorphans and their *Caballeronia* symbionts.

## supplementary material

10.1099/mgen.0.001333 Uncited Supplementary Material 1.

10.1099/mgen.0.001333 Uncited Supplementary Material 2.

## Abbreviations

iPBE: insect-associated plant beneficial and environmental clade
NCBI: National Center for Biotechnology Information
