# Supplementary material for: Inferior Caballeronia symbiont lacks conserved symbiosis genes
Source: Microb Genom. 2024 Dec 16;10(12):001333. doi: 10.1099/mgen.0.001333 (PMC11893276; doi:10.1099/mgen.0.001333)

Supplemental figures

Fig. S1. Burkholderiaceae whole-genome maximum likelihood phylogenetic tree. The phylogeny of was constructed using Realphy to compare whole genomes. Genera and clades used in analyses are highlighted. The tree was rooted with outgroup *Bordetella bronchiseptica.* Nodes with less than 50% support are collapsed. Scale bar indicates substitutions per site.

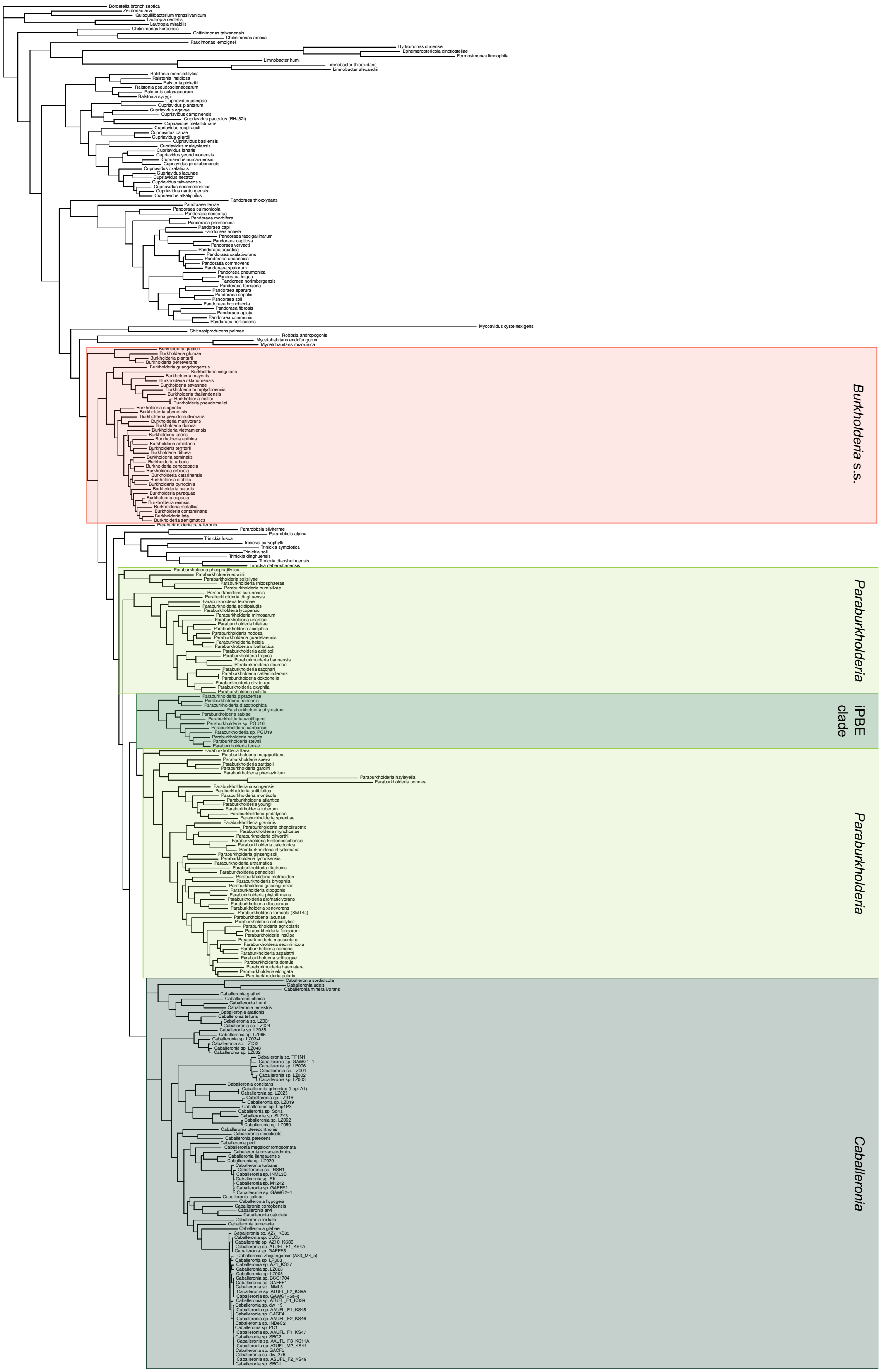
Burkholderia s.s.
Paraburkholderia
iPBE clade
Paraburkholderia
Caballeronia

Fig S2. The most likely conformations for the unique proteins, identified by Alphafold 2. Gene accession numbers are listed for each protein followed by the likelihood of the model's accuracy. The color indicates the per-residue confidence score (pLDDT) which falls between 0 and 100. Regions below 50 pLDDT may be inaccurate.

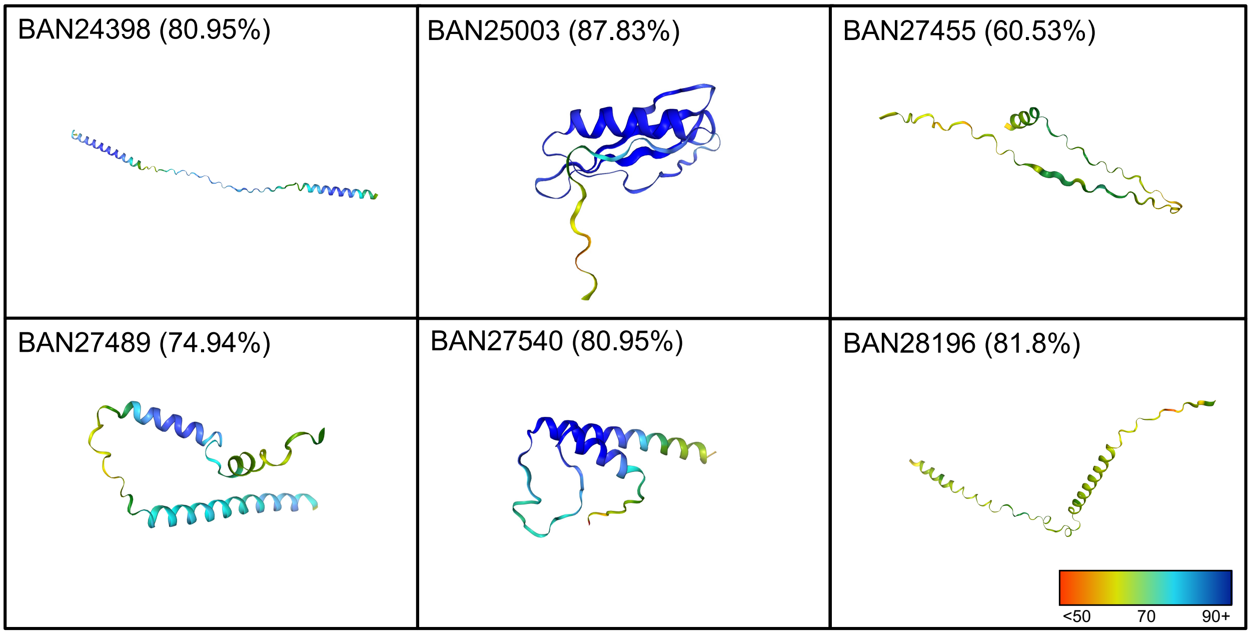

Fig. S3. *Caballeronia* whole-genome maximum likelihood phylogenetic tree. The phylogeny of was constructed using Realphy to compare whole genomes. Strains used in host fitness comparison analyses are highlighted in red. The tree was rooted with outgroup *Trinickia symbiotica.* Nodes with less than 50% support are collapsed. Scale bar indicates substitutions per site.

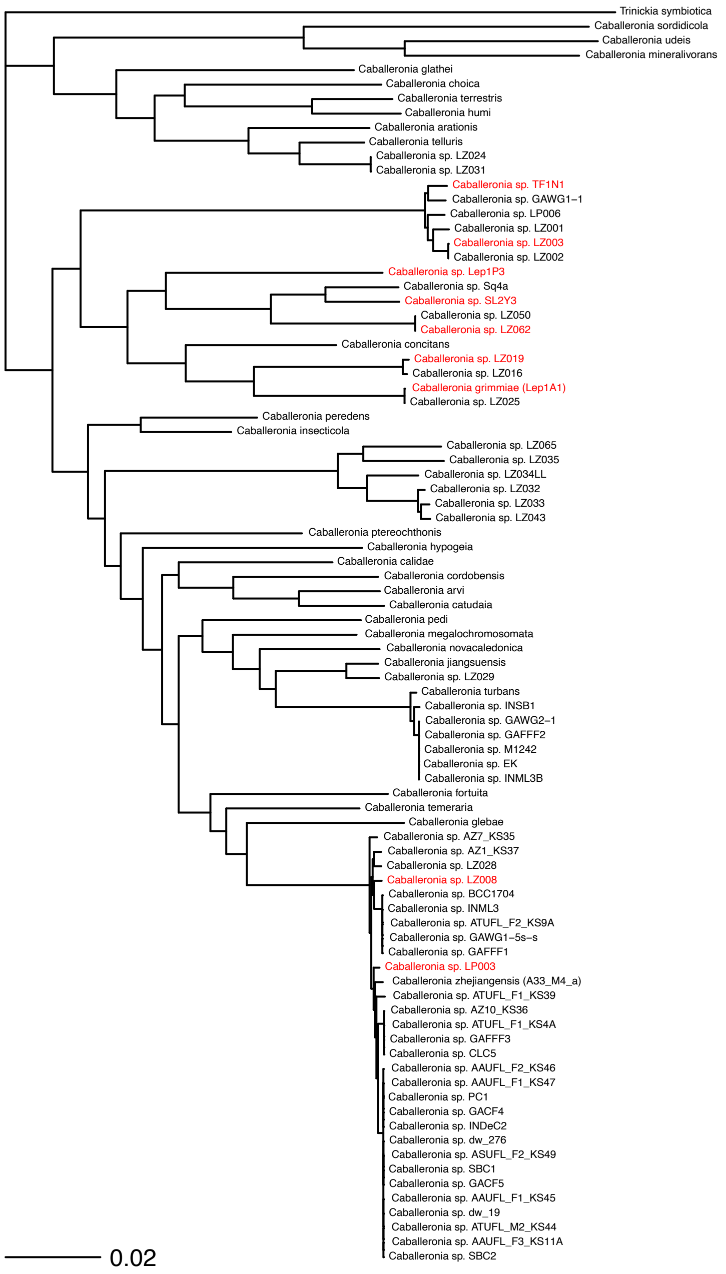

Supplement: Uncited Supplementary Material 1. [file mgen-10-01333-s001.pdf]
